# Enhanced autophagy in colorectal cancer stem cells does not contribute to radio-resistance

**DOI:** 10.18632/oncotarget.8972

**Published:** 2016-04-25

**Authors:** Chen Yan, Lan Luo, Shinji Goto, Yoshishige Urata, Chang-Ying Guo, Hanako Doi, Kaio Kitazato, Tao-Sheng Li

**Affiliations:** ^1^ Department of Stem Cell Biology, Nagasaki University Graduate School of Biomedical Sciences, Nagasaki, Japan; ^2^ Department of Thoracic Surgery, Jiangxi Cancer Hospital, Nanchang, PR China; ^3^ Division of Molecular Pharmacology of Infectious Agents, Department of Molecular Microbiology and Immunology, Graduate School of Biomedical Sciences, Nagasaki University, Nagasaki, Japan

**Keywords:** cancer stem cells, autophagy, radio-resistance

## Abstract

Autophagy, an essential catabolic pathway of degrading cellular components within the lysosome, has been found to benefit the growth and therapeutic resistance of cancer cells. In this study, we investigated the role of autophagy in the radio-sensitivity of cancer stem cells. By separating CD44^+^/CD133^+^ cancer stem cells from parental HCT8 human colorectal cancer cells, we found a significantly higher level of autophagy in the CD44^+^/CD133^+^ cells than in the parental cells. Exposure to 5 Gy of γ-ray significantly damaged both CD44^+^/CD133^+^ cells and parental cells, but the radiation-induced damage did not differ between the groups. Unexpectedly, autophagy was not significantly induced by radiation exposure in the CD44^+^/CD133^+^ cells and parental cells. The inhibition of autophagy by the silencing of ATG7, a factor required for autophagy at the stage of autophagosome precursor synthesis, did not significantly change the growth and radiation-induced damage in both CD44^+^/CD133^+^ cells and parental cells. Although an enhanced basic level of autophagy was found in the CD44^+^/CD133^+^ cancer stem cells, our data suggest that the canonical autophagy in cancer cells plays few roles, if any, in radio-sensitivity.

## INTRODUCTION

Autophagy is an essential catabolic pathway that degrades proteins or other cellular components within the lysosome [1]. The physiologic function of autophagy is well known to maintain homeostasis in mammalian cells. Autophagy also acts as a survival mechanism for cancer cells in response to various stresses during tumor progression and radiotherapy or chemotherapy [2–4]. Because the inhibition of autophagy has been experimentally demonstrated to restore therapeutic sensitivity and enhance cancer cell death [2–6], several autophagy inhibitors have been clinically tested in cancer patients [7, 8]. Although these early-phase clinical trials have reported positive data of antitumor benefits [7, 8], the role of autophagy in mediating the progression and therapeutic resistance of cancer remains unclear.

In past decades, a stem cell-like subpopulation known as “cancer stem cells” (CSCs) has been found in various types of malignant tumors. Although lacking consensus, some cell surface markers for hematopoietic stem cells, such as CD44 and CD133, have been popularly used for the identification/purification of CSCs [9, 10]. These CSCs are thought to demonstrate therapeutic resistance and play critical roles in the recurrence and metastasis of cancer [9]. Autophagy has also been highly observed in CSCs [11–13], and the induction of autophagy can contribute to radio-resistance in glioma stem cells [11]. Furthermore, the relative lower reactive oxygen species (ROS) level in CSCs may confer the resistance to irradiation [14]. However, it has no consensus about the consequence relation between autophagy and radio-resistance of cancer cells [15].

Using the HCT8 human colorectal cancer cell line, we isolated and purified CD44^+^/CD133^+^ CSCs from parental cells and then compared the autophagy and radio-sensitivity between cells. Although a higher basal level of autophagy was observed in the CD44^+^/CD133^+^ CSCs than in the parental cells, there was no significant difference in radio-sensitivity between the CD44^+^/CD133^+^ CSCs and parental cells following exposure to 5 Gy of γ-ray. Furthermore, autophagy was not significantly induced by irradiation, and the inhibition of autophagy did not change the radio-sensitivity in both of the CD44^+^/CD133^+^ CSCs and parental cells.

## RESULTS

### Enhanced autophagy in CD133^+^/CD44^+^ CSCs

**Figure 1:**
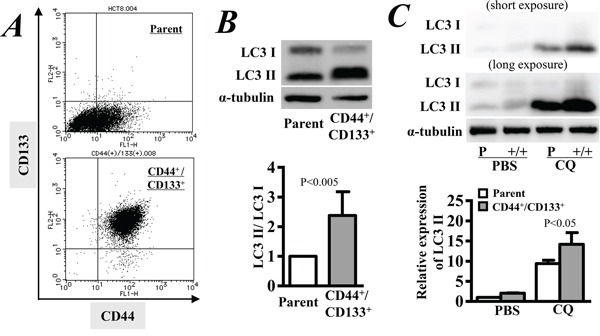
Autophagy activity in CD44^+^/CD133^+^ cancer stem cells **A.** CD44^+^/CD133^+^ cancer stem cells were purified from the HCT8 human colorectal cancer cell line, and the purity was confirmed by flow cytometry. **B.** Western blot analysis was used to detect the expression levels of LC3-I and LC3-II, and the LC3-II/LC3-I ratio represents the extent of autophagy. **C.** Autophagic flux by inferring with LC3-II turnover by Western blotting in the presence and absence of a lysosomal inhibitor. Cells were incubated with 50 μM chloroquine (CQ) for 4 hrs. Parental cells without any treatment were used as the control of relative expression. PBS: Phosphate-buffered saline. The data are represented as the means ± SD from three independent experiments.

We isolated the CD133^+^/CD44^+^ CSCs from parental HCT8 cells, and the purity was more than 95% by flow cytometry analysis (Figure 1A). The expression of CD133 and CD44 in the purified CSCs was stable for at least 45 days after culture. Western blotting detected more extensive expression of LC3-II in CD133^+^/CD44^+^ CSCs than in parental cells (Figure 1B). Quantitative analysis showed that the expression ratio of LC3-II to LC3-I, a parameter generally used to determine the extent of autophagy, was approximately two-fold higher in CD133^+^/CD44^+^ CSCs than in parental cells (p < 0.005, Figure 1B), indicating an intrinsic high basal level of autophagy in CD133^+^/CD44^+^ CSCs. We also measured autophagic flux by inferring LC3-II turnover in the presence and absence of chloroquine (CQ). Western blotting showed that the expression of LC3-II was increased in both cells after 4 hrs of incubation with 50 μM CQ, but the relative expression of LC3-II was significantly higher in CD133+/CD44+ CSCs than in parental cells (p < 0.05, Figure 1C). Thus, there was a higher autophagic flux level in CD133^+^/CD44^+^ CSCs than in parental cells.

### Radiation-induced cell damage did not differ between CD133^+^/CD44^+^ CSCs and parental cells

**Figure 2:**
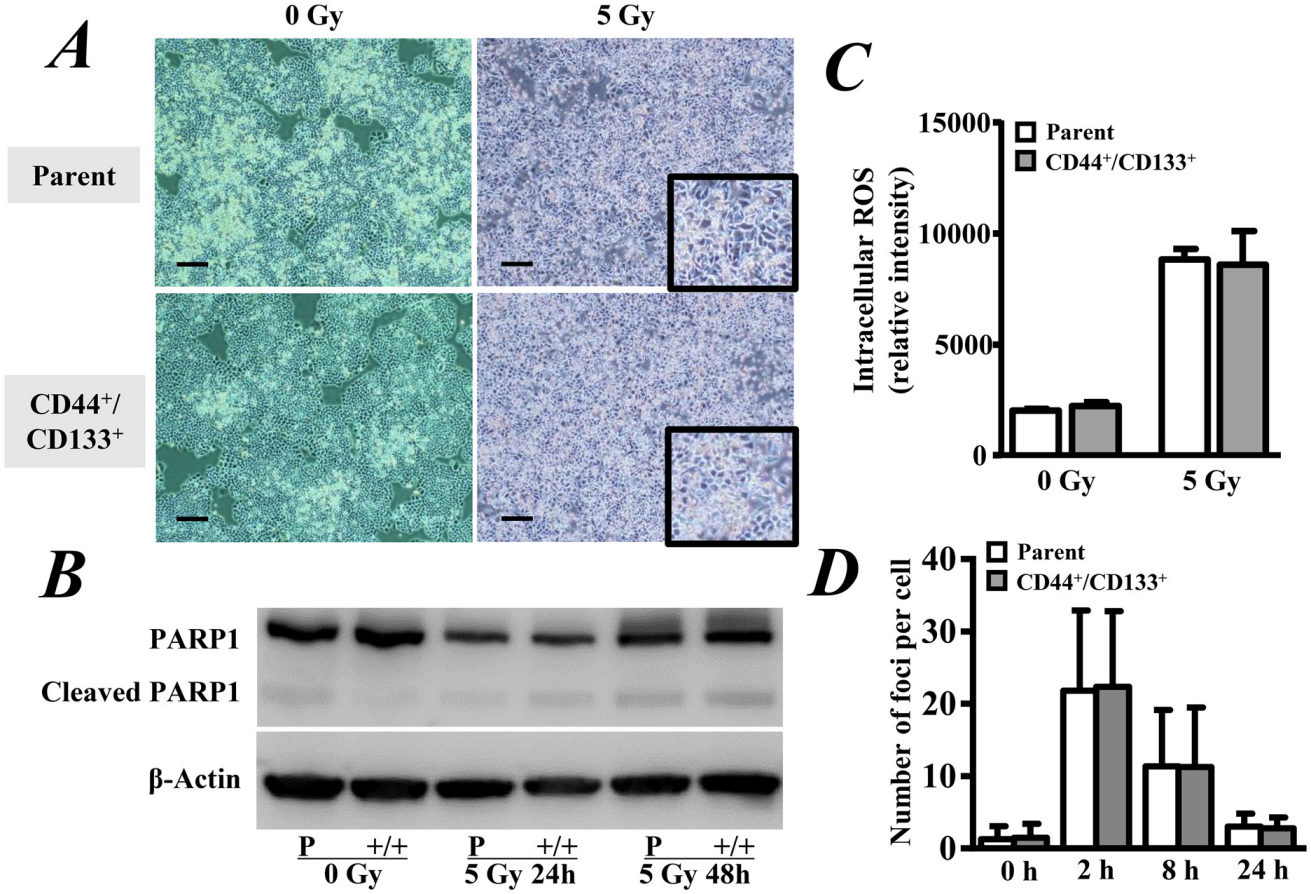
Radiation-induced cell damage **A.** CD44^+^/CD133^+^ cancer stem cells and parental HCT8 human colorectal cancer cells were exposed to 5 Gy of γ-ray followed by incubation for another 2 days. The growth of cells was observed under a microscope with 40-fold magnification. Scale bar, 200 μm. **B.** Western blot analysis of the expression level of cleaved PARP1 in cells. **C.** Cells were labeled with 10 μM H_2_DCFDA for 10 min and then exposed to 5 Gy of γ-ray. The ROS level was evaluated by measuring the fluorescence intensity within cells. **D.** Cells were exposed to 5 Gy of γ-ray and then fixed at the indicated times. The number of γ-H2AX foci in each cell was counted under fluorescence microscopy, and the mean number of γ-H2AX foci per cell was calculated. The data are represented as the means ± SD from three independent experiments.

We exposed the cells to 5 Gy of γ-ray and then continued to incubate the cells for another 2 days. Both of the CD133^+^/CD44^+^ CSCs and parental cells continued growing slowly even after radiation exposure (Figure 2A). Western blotting showed a comparable level of cleaved PARP1 between the CD133^+^/CD44^+^ CSCs and parental cells, although radiation obviously induced the expression of cleaved PARP1 in both cell types (Figure 2B).

The ROS level was quantitatively measured by the fluorescence intensity after loading with H_2_DCFDA. Although radiation exposure significantly increased the ROS level in both the CD133^+^/CD44^+^ CSCs and parental cells 15 min after 5 Gy of γ-ray exposure (p < 0.05, Figure 2C), there was no significant difference between the two cell types (p > 0.05, Figure 2C).

We also investigated the DNA damage by measuring γ-H2AX foci after radiation. All of the cells showed some γ-H2AX foci within the nucleus. Although the number of γ-H2AX foci per cell was significantly increased in the CD133^+^/CD44^+^ CSCs and parental cells at 2 and 8 hrs, respectively, after radiation (p < 0.05, Figure 2D), there was no significant difference between the two types of cells (p > 0.05, Figure 2D).

### Autophagy did not significantly contribute to radio-resistance

**Figure 3:**
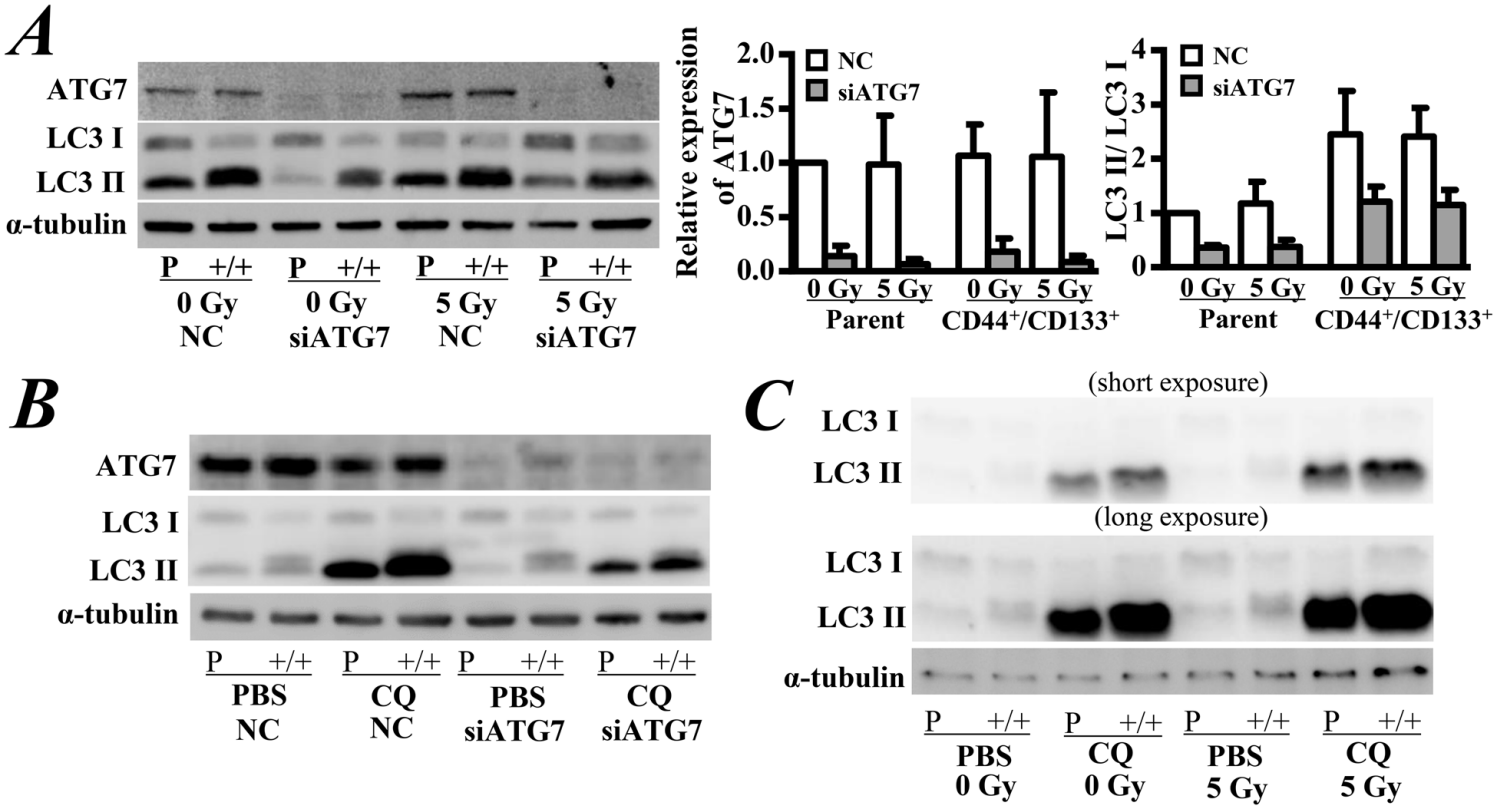
Western blot analysis of the expression of ATG7 and LC3 **A.** Cells were treated with ATG7 siRNA and then incubated for 2 days. After autophagy was inhibited, cells were exposed to 5 Gy of γ-ray followed by incubation for another 2 days. The representative western blot shows the expression of ATG7 and LC3 in cells. Quantitative analysis of the LC3-I/LC3-II ratio and relative expression level of ATG7. Parental cells without any treatment were used as the control of relative expression. **B.** Autophagic flux was measured after ATG7 knockdown. Cells were incubated with 50 μM chloroquine (CQ) for 4 hrs. **C.** Autophagic flux was measured after irradiation. Parental cells without any treatment were used as the control of relative expression. NC: negative control siRNA. PBS: Phosphate-buffered saline. The data are represented as the means ± SD from three independent experiments.

To further confirm the role of autophagy on the cytotoxic effect with or without radiation exposure, we silenced the expression of ATG7, a factor required for autophagy at the stage of autophagosome precursor synthesis. Transfection with ATG7 siRNA significantly decreased the protein level of ATG7 and the ratio of LC3-II/LC3-I expression in both CD133^+^/CD44^+^ CSCs and parental cells (p < 0.05, Figure 3A). Although a higher ratio of LC3-II/LC3-I expression was observed in CD133^+^/CD44^+^ CSCs than in parental cells, exposure to 5 Gy of γ-ray did not significantly change the expression of ATG7 and the ratio of LC3-II/LC3-I expression in all cells (Figure 3A). After knockdown of ATG7, the autophagic flux level was decreased in both of the CD133^+^/CD44^+^ CSCs and parental cells (Figure 3B). However, the autophagic flux level did not change after radiation in the two types of cells (Figure 3C).

**Figure 4:**
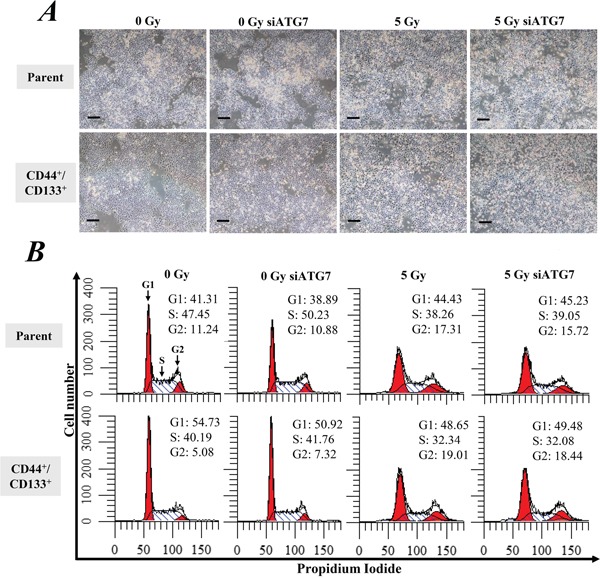
Cell growth and the cell cycle Cells were treated with ATG7 siRNA and then incubated for 2 days. After autophagy was inhibited, cells were exposed to 5 Gy of γ-ray followed by incubation for another 2 days. **A.** Cell growth was observed under a microscope with 40-fold magnification. Scale bar, 200 μm. **B.** The cell cycle was measured by PI staining.

**Figure 5:**
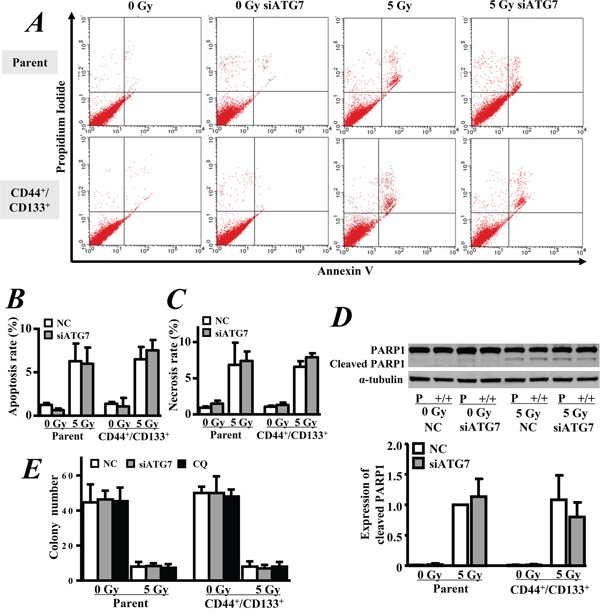
Apoptosis and clonogenic survival Autophagy was inhibited by ATG7 siRNA for 2 days or 50 μM chloroquine (CQ) for 4 hrs. **A.** The cell apoptosis was measured by Annexin V/PI staining. **B.** Quantitative analysis of the apoptosis rate. **C.** Quantitative analysis of the necrosis rate. **D.** Western blot analysis of the expression of cleaved PARP1. Parental cells exposed to 5 Gy of γ-ray were used as the control of relative expression. **E.** Clonogenic survival assay. NC: negative control siRNA. The data are represented as the means ± SD from three independent experiments.

We further observed the growth and apoptosis of cells for another 2 days after exposure to 5 Gy of γ-ray. Both of the CD133^+^/CD44^+^ CSCs and parental cells continued growing slowly even after radiation exposure (Figure 4A). However, the cells at G2-phase were obviously increased in both of the CD133^+^/CD44^+^ CSCs and parental cells after irradiation, indicating radiation-induced cell cycle arrest (Figure 4B). Annexin V/PI staining showed that the exposure to 5 Gy of γ-ray also significantly increased cell apoptosis (p < 0.05, Figure 5A, 5B) and necrosis (p < 0.05, Figure 5A, 5C). However, the inhibition of autophagy by ATG7 siRNA did not significantly change cell apoptosis and necrosis. Similarly, Western blot analysis showed that radiation significantly enhanced the expression of cleaved PARP1 (p < 0.05 *vs.* 0 Gy, Figure 5D), but the inhibition of autophagy by ATG7 siRNA did not significantly change the expression of cleaved PARP1 in all cells, and ATG7 siRNA even slightly decreased the expression of cleaved PARP1 in the CD133^+^/CD44^+^ CSCs with radiation exposure (Figure 5D). A clonogenic assay showed that radiation significantly decreased the number of colonies between CD133^+^/CD44^+^ CSCs and parental cells (p < 0.05, Figure 5E), but there was no significant difference between the two types of cells. Furthermore, the inhibition of autophagy by ATG7 siRNA or chloroquine did not significantly change the colony formation ability in both cell types (p > 0.05, Figure 5E).

## DISCUSSION

Colorectal cancer is the third most common cancer and fourth most common cause of cancer death globally [16]. In addition to colorectal surgery, additional chemotherapy or radiotherapy may prove beneficial as well [16]. Unfortunately, only approximately 20% of colorectal cancers achieve complete pathologic responses to chemotherapy, and radiotherapy seems to be beneficial in few cases, if any [17]. Therefore, many efforts have been made to improve the radio-sensitivity of colorectal cancer [18–20]. Because autophagy is generally considered a pro-survival mechanism of cells to stresses [2, 11, 21], the combination of irradiation with autophagy inhibition has also been clinically tested to improve the sensitivity of killing cancer cells [22, 23]. Complex factors, including the enhanced DNA damage response, ROS scavenging, autophagy, activation of developmental pathways, and microenvironmental stimuli, seem to be associated with the radio-resistance of cancer [24–28]. However, the precise mechanism underlying the radio-resistance of colorectal cancer remains incompletely understood.

Different methods have been used to identify the CSCs in colorectal cancer [29–31], and the isolated subpopulation of CD44^+^/CD133^+^ cells from human colorectal cancer has been confirmed to be characterized as CSCs [31]. Because CSCs have been found to be resistant to radiation [32], we tried to uncover the role of autophagy in radio-resistance by purifying the CD44^+^/CD133^+^ CSCs from the HCT8 human colorectal cancer cell line. These purified CD44^+^/CD133^+^ CSCs showed higher autophagy than the parental cells. Although it has been reported that autophagy can reduce the ROS level under oxidative stress [33], our data showed comparable ROS levels between the CD44^+^/CD133^+^ CSCs and parental cells.

Apoptosis is considered the principal cell death pathway elicited by radiotherapy, and radiotherapy may employ ROS to eradicate cancer cells [34]. DNA double-strand breaks (DSBs) represent important radiation-induced lesions, and impaired DSB repair provides the best available correlation with radio-sensitivity [35]. Although radiation exposure significantly increased the ROS level and damaged the cells, our data showed comparable radio-sensitivity between the CD44^+^/CD133^+^ CSCs and parental cells. Surprisingly, radiation did not significantly induce autophagy in both cell types, even if we exposed the cells to a higher dose (up to 20 Gy, data not shown). To further confirm the role of autophagy on radio-sensitivity, we tried to inhibit the autophagy pathway by silencing ATG7 and chloroquine [15, 36]. Again, the inhibition of autophagy in both cell types did not change the cell growth and radio-sensitivity.

It remains unknown why autophagy was not significantly induced by radiation and contributed to radio-sensitivity in our study. Actually, the role of autophagy in the radio-resistance of cancer cells is controversial [24, 37]. It has been reported that autophagy plays a cytoprotective role in the radiation of glioma stem cells [5, 11]. However, another study found that the radio-resistance of cancer cells is canonical autophagy independent [15]. More recently, Gewirtz has proposed that autophagy may functionally serve cytoprotective, cytostatic, cytotoxic, and nonprotective roles, depending on the cell types and conditions, as well as various stresses [38]. Therefore, enhanced autophagy activity in these CD44^+^/CD133^+^ CSCs may only serve in a nonprotective role in radiation.

In conclusion, enhanced autophagy activity was observed in the CD44^+^/CD133^+^ CSCs purified from the HCT8 human colorectal cancer cell line. Based on the data from the present study, autophagy plays few roles, if any, in the radio-resistance of cancer cells. These results provide new insights for understanding the role of autophagy in the radio-resistance of CSCs.

## MATERIALS AND METHODS

### Cell culture

The HCT8 human colorectal cancer cell line was used for experiments. Cells were maintained in RPMI 1640 medium (Wako, Japan) with 10% fetal bovine serum and 1% penicillin/streptomycin (Gibco, UK). Cells were cultured at 37°C in a humidified atmosphere of 5% CO_2_ and 95% air.

### Purification of CD133^+^/CD44^+^ CSCs

A single-cell suspension of HCT8 cells was incubated with magnetic microbeads-conjugated with the mouse anti-human CD44 monoclonal antibody (Miltenyi Biotec, USA) for 30 min. After washing, the CD44^+^ cells were separated using the Magnetic Cell Sorting system (autoMACS; Miltenyi Biotec, USA) [39]. The purified CD44^+^ cells were expanded for 14 days by culture and then harvested as a single-cell suspension to be incubated with magnetic microbeads-conjugated mouse anti-human CD133 monoclonal antibody (Miltenyi Biotec, USA) for 30 min. After washing, the CD133^+^ cells were separated using the Magnetic Cell Sorting system as described above. This two-step isolation enabled us to obtain a sufficient number of CD44^+^/CD133^+^ CSCs for the experiment.

To verify the purity of the isolated CD133^+^/CD44^+^ CSCs, cells were stained according to the supplied antibody protocols. Mouse anti-Human CD133/1 (Clone: AC133)-PE and mouse anti-human CD44 (Clone: DB105)-FITC (Miltenyi Biotec, USA) were used, and flow cytometry analysis was performed using a FACSCalibur instrument (Becton Dickinson).

### Radiation exposure

Cells in culture dishes were exposed to 0 or 5 Gy (^137^Cs source in a PS-3100SB γ-ray irradiation system; 1 Gy/min; Pony Industry Co., Ltd. Osaka, Japan) [40]. The distance from the radiation source to the bottom of the dishes was set at 401.9 mm.

### Detection of intracellular ROS level

We measured the intracellular ROS level based on the oxidation of 2′, 7′-dichlorodihydrofluorescein diacetate (H_2_DCFDA, Molecular Probes Inc., USA) to form the fluorescent compound 2′, 7′-dichlorofluorescein (DCF). Briefly, cells were loaded with 10 μM H_2_DCFDA for 10 min. After washing, cells were exposed to 5 Gy γ-ray and then incubated at 37°C for 15 minutes. The fluorescence intensity was measured by a plate reader (VICTOR™ X3 Multilabel Plate Reader; PerkinElmer Inc., Waltham, Massachusetts, USA).

### Immunofluorescence for γ-H2AX

Immunofluorescence for γ-H2AX, a DNA damage and repair marker, was also evaluated. Briefly, cells were exposed to 0 or 5 Gy of γ-ray. At the indicated times after irradiation, cells were washed with cold PBS and fixed with 4% formalin for 10 min at room temperature. After washing three times with PBS, cells were incubated with a rabbit anti-human γ-H2AX polyclonal antibody (1:500 dilution, Abcam) overnight at 4°C. After washing three times with PBS, cells were incubated with an Alexa Fluor® 488 conjugated goat anti-rabbit secondary antibody (1:250 dilution; Molecular Probes Inc., USA) at room temperature for 2 hrs in the dark. The immunofluorescence for γ-H2AX in cells was examined on an Olympus fluorescent microscope, and the number of γ-H2AX foci per cell was counted under the microscope with 400-fold magnification. The mean number of γ-H2AX foci from more than 50 cells was calculated for statistical analysis.

### RNA interference

siRNA specific to ATG7 (#6604S) and a control siRNA (#6568S) were obtained from SignalSilence (Cell Signaling Technology, Inc.). We seeded cells on 6-well plate (2×10^5^ cells/well) 16 hrs before siRNA transfection. Transfection was performed using LipofectAMINE 3000 (Invitrogen) according to the manufacturer's instructions. A total of 100 nmol/L of siRNA duplex was used for each transfection.

### Western blotting

Following the radiation exposure as indicated, cells were washed with PBS and lysed at 4°C in lysis buffer. Insoluble material was removed by centrifugation at 15,000 × g for 15 min. Total proteins were separated by SDS-PAGE gels and then transferred to 0.22-μm PVDF membranes (Bio-Rad) as described previously [39]. After blocking, the membranes were incubated with rabbit anti-human LC3 polyclonal antibody (1:500 dilution; Novus Biologicals), rabbit anti-human PARP1 polyclonal antibody (1:1000 dilution; Cell Signaling Technology), rabbit anti-human ATG7 polyclonal antibody (1:500 dilution; Cell Signaling Technology), mouse anti-human α-tubulin antibody (1:4000 dilution; Cell Signaling Technology), mouse anti-human β-actin (1:5000 dilution; Sigma-Aldrich), followed by the appropriate horseradish peroxidase-conjugated secondary antibodies (Dako, Japan). The expression was visualized using an enhanced chemiluminescence detection kit, and semi-quantitative analysis was performed by measuring the density of the bands using ImageQuant LAS 4000 mini (GE Healthcare Life Sciences) [39].

### Cell cycle analysis

Cells were harvested as a single-cell suspension and fixed in cold 70% ethanol for 30 min at 4°C. After washing with PBS, 0.5 mL of FxCycle™ PI/RNase Staining Solution (Thermo Fisher Scientific, Inc.) was added for incubation. Flow cytometry analysis was performed using a FACSCalibur instrument (Becton Dickinson).

### Apoptosis assays

Cells were seeded in 6-well culture plates (2×10^5^ per well). After treatment, cells were harvested at the indicated time points. FITC-Annexin V and Propidium Iodide staining was performed using the Annexin V-FITC Apoptosis Detection Kit (Beckman Coulter, Inc., USA) according to the manufacturer's instructions, followed by flow cytometry analysis using a FACSCalibur instrument (Becton Dickinson).

### Clonogenic survival assay

To evaluate the colony-forming ability after irradiation, we seeded cells into 6-well plates at a density of 100 cells/well. After overnight incubation, cells were exposed to 5 Gy of γ-ray, and the formation of colonies was quantified at 7 days after irradiation exposure. Colonies with over 50 cells were counted under an Olympus microscope.

### Statistical analyses

Data are represented as the means ± SD. The statistical significance was determined by one-way analysis of variance (ANOVA) followed by Tukey's test (Dr. SPSS II, Chicago, IL). A p value less than 0.05 was accepted as statistically significant.
